# The Head Tracks and Gaze Predicts: How the World’s Best Batters Hit a Ball

**DOI:** 10.1371/journal.pone.0058289

**Published:** 2013-03-13

**Authors:** David L. Mann, Wayne Spratford, Bruce Abernethy

**Affiliations:** 1 MOVE Research Institute Amsterdam, Faculty of Human Movement Sciences, VU University, Amsterdam, The Netherlands; 2 Institute of Human Performance, University of Hong Kong, Pokfulam, Hong Kong SAR; 3 Movement Science - Biomechanics, Australian Institute of Sport, Canberra, ACT, Australia; 4 Cricket Australia Centre of Excellence, Brisbane, Queensland, Australia; 5 School of Human Movement Studies, University of Queensland, Brisbane, Queensland, Australia; The Australian National University, Australia

## Abstract

Hitters in fast ball-sports do not align their gaze with the ball throughout ball-flight; rather, they use predictive eye movement strategies that contribute towards their level of interceptive skill. Existing studies claim that (i) baseball and cricket batters cannot track the ball because it moves too quickly to be tracked by the eyes, and that consequently (ii) batters do not – and possibly cannot – watch the ball at the moment they hit it. However, to date no studies have examined the gaze of truly elite batters. We examined the eye and head movements of two of the world’s best cricket batters and found both claims do not apply to these batters. Remarkably, the batters coupled the rotation of their head to the movement of the ball, ensuring the ball remained in a consistent direction relative to their head. To this end, the ball could be followed if the batters simply moved their head and kept their eyes still. Instead of doing so, we show the elite batters used distinctive eye movement strategies, usually relying on two predictive saccades to anticipate (i) the location of ball-bounce, and (ii) the location of bat-ball contact, ensuring they could direct their gaze towards the ball as they hit it. These specific head and eye movement strategies play important functional roles in contributing towards interceptive expertise.

## Introduction

Spectators marvel at the ability of skilled hitters in fast ball-sports. Batters produce remarkably precise visually guided movements to hit balls travelling at speeds that test the limit of human visual-motor control [1], [2], and they hit the ball despite late and unexpected deviations in its flight-path [3]. Examining elite performers of these tasks provides a unique opportunity to better understand how interceptive actions are controlled and performed, though to this point, there have been very few opportunities to scientifically examine the exquisite hitting skills of truly elite performers in-situ. Here we examine the visual-motor performance of two of the most accomplished cricket batters to have played the game, and show that they use distinct strategies to control their gaze and head to underpin their batting success – strategies that are not evident with less accomplished players.

The edict to ‘keep your eye on the ball’ is one of the oldest coaching mantras in sport, yet surprisingly a number of studies suggest it may be impossible to do so. Players do not align their central (foveal) gaze with the ball throughout its flight-path across a range of different sports, including in baseball [4], [5], cricket [6], [7], table tennis [8], and squash [9]. In particular, central vision has been shown to lag behind the ball before batters can hit it, and as a result, batters might not watch the ball at the moment it is hit [4], [5], [7]. In conditions where it may be difficult to track the flight-path of the ball, *predictive saccades* are commonly used to move gaze ahead of the ball, or to catch up with it [4], [6], [7]. It is not clear why hitters do not track the ball throughout the entirety of its flight-path, though it is often said that the ball moves too quickly for the eyes to be able to track it [4], [7], [8].

Importantly, eye movement strategies are associated with skill in batting. Bahill and LaRitz [4] demonstrated that a moderate-level major league baseballer was able to track a ball (moving along a string) for a longer period of time than lesser skilled players could. More recently, Land and McLeod [7] recorded the eye movements of three cricket batters with varying levels of batting skill (state/provincial professional, high-level amateur, low-level amateur) and found a systematic relationship between gaze and batting skill. In their study, batters were found to track the ball for a short period after ball-release before making a predictive saccade to anticipate where the ball would bounce (in cricket, like in tennis, a hitter typically hits the ball after it has bounced). Critically, the predictive saccade occurred *earlier* as the skill level of the batter increased, reflecting a superior ability to predict the future landing point of the ball. Crucially, this highlights that there may be some functional advantage in producing a predictive saccade when hitting a moving target. In showing that eye movement strategies contribute to skill in hitting, these studies have been influential at both an applied and theoretical level. Unfortunately, though, there are two clear limitations. First, there is some doubt that these findings represent those that would occur in a natural setting, as the experimental tasks have been simplified (using a ball on a string or a bowling machine) and don’t necessarily represent those performed in the natural environment [10]. Second, these studies have not considered the visual-motor capabilities of truly elite batters. By implying that a linear relationship exists between visual skill and batting ability, their findings may misrepresent the true ability of elite performers.

One particular issue worthy of consideration is whether batters watch the ball at the moment they hit it. Scientific studies of visual-motor control report that batters do not – and perhaps cannot – watch the ball when it is hit [4], [5], [7]. Yet, this is at odds with the anecdotal reports of some elite batters [e.g., 4]. For example, Justin Langer, a recently retired international batter (and more recently, the Australian Batting Coach) found the concept of not watching the ball hit the bat as *unbelievable*, as he clearly describes seeing markings on the ball as it makes contact with the bat (personal communication, 6/03/11). Further, a current international player, one of the top five international run-scorers of all time, reports that one of his key aims when batting is to *watch the ball come out from underneath my bat* when he hits it (personal communication, 9/18/11). It is highly unlikely that either of these tasks would be possible unless the ball was fixated using central vision at the moment of bat-ball contact. This highlights the need for studies of transcendent experts in hitting sports as it suggests that either the current scientific account of the visual-motor control of skilled batting is wrong, or that elite batters possess a misconstrued understanding of their perceptual and action capabilities when batting [11]. Rather than being a trivial point, this issue has important theoretical implications. The inability to watch the ball at bat-ball contact has been interpreted as evidence that hitting is highly predictive and relies on a pre-programmed movement strategy [7], [12]. According to this theory the batter must predict the future location of the ball and produce a pre-programmed hitting action to account for the considerable time-delay between the perception of ball-flight and the time taken to produce the appropriate hitting response [13], [14]. In this sense, late ball-flight information may be superfluous for the performance of the hitting action [7]. Evidence to the contrary would question this supposition, and leave open the possibility that late ball-flight information could be used to modify the interceptive action based on continuous visual feedback from the position of the ball [e.g., 1,15,16].

A growing body of work highlights that an examination of the coordinating perceptual system (incorporating the eyes and head) can provide a clearer representation of visual-motor control than from an examination of gaze alone [e.g., 17,18]. In the case of hitting tasks, for example, elite athletes in sports such as baseball and tennis are often said to maintain a ‘still’ head throughout their hitting actions [19], yet surprisingly very few researchers have sought to qualitatively establish what this means, and if it makes any contribution towards the development of expertise. Head movement can play an important role when tracking a fast-moving target [9], but importantly, there is good reason to believe that coupling the movement of the head to a target may provide a functional advantage when producing an interceptive action. For example, catching studies show that observers mainly rotate their eyes when they stand and passively watch where a fly-ball will land, but they rotate their eyes *and head* when they try to catch it [20], [21]. When catching, the head movement ensures that the egocentric point of reference changes; that is to say, the head moves to keep the ball in a more consistent direction relative to the head. This concept is consistent with the notion that visual-perceptual and visual-motor tasks rely on different types of visual information, in particular, observers in visual-perceptual tasks gather information allocentrically (i.e., object-centred relative to the surrounding environment), while performers of visual-motor tasks gather information egocentrically (i.e., consistent according to self-centred coordinates) [e.g., 22,23].

Further evidence exists to suggest that the head may move to keep a target in a consistent egocentric frame of reference in aiming tasks. Skilled basketballers direct or *anchor* their head towards the ring when performing jump shots [24]; as a result, they spend a longer period of time with the ring kept in a consistent head-centred egocentric direction as the shot is prepared. Similarly, a skilled racing car driver has been shown to couple his head direction to the rate of car rotation to ensure that the targeted location was kept in line with the direction of the head [25]. This finding implies that the driver used a common mechanism to control his head direction and the steering of the race car. Collectively these findings suggest that the ability to maintain the target in a consistent head-centred egocentric direction may be an important element of skill in interceptive tasks. In the case of hitting sports, it raises the possibility that an important component of expertise may be an ability to couple the movement of the head to the flight-path of the ball, ensuring the ball is kept in a consistent egocentric direction relative to the batter’s head. In other words, the batter may move his or her head in a manner that keeps the ball in the same direction within the local head-centred coordinate system. If the ability to couple head movement to the ball is an important element of skill in batting, then it is reasonable to expect that a commensurate relationship should exist between head-ball coupling and batting skill.

In this study we took the unique opportunity to measure the eye and head movements of two of the world’s best cricket batters: one was a recently retired opening batsman who played more than 100 test matches for Australia, and the other was a batsman in the Australian test side at the time of testing. We compared the visual-motor performance of these two *elite* batters with two competent but considerably less-skilled *club* level batters. We expected to find systematic differences in the eye and head movements according to their level of batting skill. Specifically, we hypothesised that the elite batters would (i) utilise eye movement strategies that enabled them to observe the ball at the moment it was hit, and that the elite batters would (ii) couple the rotation of their head to the movement of the ball, ensuring the ball was kept in a consistent egocentric direction throughout the hitting action.

## Materials and Methods

### Participants

Two elite and two club-level cricket batters took part in the study. At the time of testing, the elite batters had both represented their country in more than 70 international test matches, averaging greater than 45 runs per innings (a feat achieved by only 43 of 2645 international players since 1877). The two club batters both played recreationally at a high-level in a domestic club competition.

### Experimental Task

Participants batted against a ProBatter ball-projection machine (ProBatter Sports, Milford, CT) which displayed life-size video-projected footage of a bowler in their approach towards the batter, and at the moment of ball-release, a ball was projected through a hole in the screen. Batters stood ≈17.7 m from the location of ball-release, with this and all other pitch and stump dimensions replicating those experienced in a match. The ProBatter machine provided three specific experimental advantages when compared to the use of real bowlers or a bowling/projection machine. First, it provided experimental control of ball delivery that would not be possible if participants were to face bowlers in-situ [26]–[28]. Second, when compared to a projection machine, the video footage of a bowler provided advance bowler-specific movement information that is known to be useful in movement coupling when batting [29], [30]. Third, the projection machine was not visible to batters, ensuring they could not see the angle of the machine to provide cues to the potential direction of ball projection [31].

Participants wore a Mobile Eye eye-tracking device (Applied Sciences Laboratories, Bedford, MA; 25 Hz) to record the direction of the head and visual gaze when batting. Footage from the Mobile Eye glasses worn by participants were recorded on a digital video (DV) recording device held within a waist pouch tightly wrapped around the lower back of participants. A radio transmitter attached to the DV recorder wirelessly transmitted the video signal so that footage could be monitored contemporaneously on a large television screen located adjacent to the testing area. Real-time monitoring was used to check for changes in the direction of the scene or eye cameras so any alterations in calibration could be immediately rectified to maximise the percentage of useful data recorded by the eye-tracking system. A DV camera (25 Hz) was located behind participants to confirm the moments of ball-release, ball-bounce, and bat-ball contact if any were not visible in the footage from the Mobile Eye scene camera.

Participants wore a cricket helmet with a small portion of the peak cut away to prevent mislocation of the scene camera by any movement of the helmet. Calibration was performed after the initial fitting of the Mobile Eye using a number of pre-determined locations within the visual scene. Recalibration was performed prior to, and following each testing condition, when the view monitored on the remote screen was deemed to have moved, or when participants felt that they had knocked the eye-tracking system.

### Procedure and Design

Participants batted against six types of trials that differed according to the location of ball-bounce. In cricket, the location of ball-bounce varies according to how close it bounces to the batter (*length*), and the direction in which the ball is headed towards (*line*). Deliveries bounced at one of three different lengths (*full*, *good*, and *short*), and two different lines (*straight* and *off*, directed towards, and away from, the batter’s body respectively). In the experiment proper, participants faced a total of 18 trials: three trials for each of the six different deliveries (full & off, full & straight, good & off, good & straight, short & off, shot & straight) presented in a randomised order. Variations in line were of no interest to the experimental examinations; rather they were included to minimise the chance of the batters predicting the location of ball-bounce based on probability alone. The ball speed (33.3 m/s or 120 km/h) was considerably faster, and more representative of real competition, when compared to speeds used in previous examinations of interceptive skill [typically 60–90 km/h or 17–25 m/s; 6,7]. Prior to testing, participants faced approximately 15–20 deliveries to familiarise themselves with the ProBatter machine, with the synthetic (artificial grass) indoor playing surface, and with the different types of deliveries they were to experience in the experiment proper. The experimental procedure received ethical approval through the University of Queensland and participants provided written informed consent to take part in the experiment.

### Data Analysis

**Figure 1:**
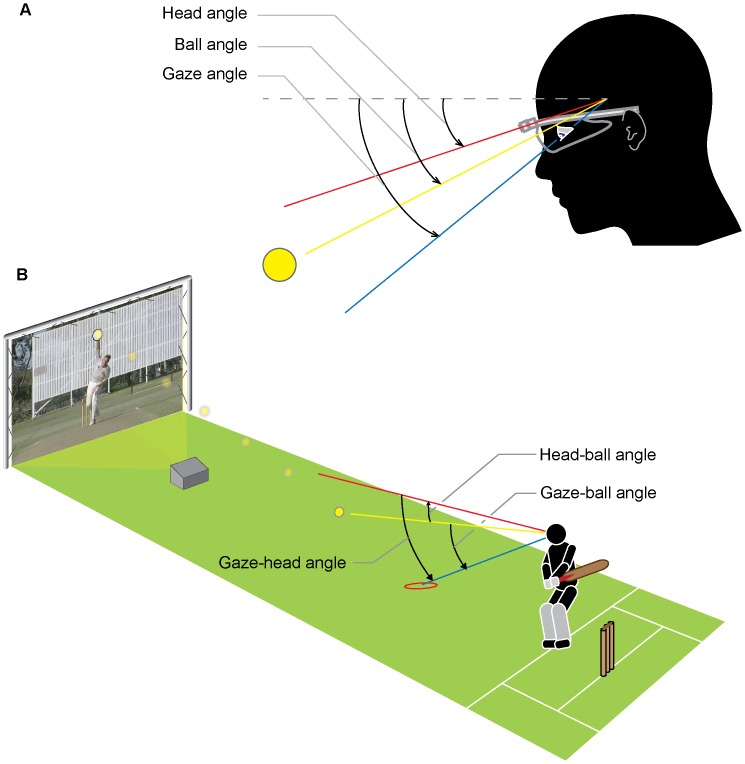
Experimental set-up and measurement of head, ball, and gaze angles. Panel A shows the three individual angles, and Panel B shows the three relative angles used to measure the movement of the eyes and head relative to the ball, and the eyes relative to the head. Individual angles are subtended by direction of the ball (yellow), gaze (blue), and head (red) at the eye (in degrees) relative to the initial direction at ball-release (dotted line shows the case where all three angles coincide at ball-release). In all cases a negative angle refers to a downward direction. Panel B shows the experimental set-up; in this case showing a positive gaze-ball angle (gaze located ahead of ball); negative head-ball angle (direction of head lagging behind ball); and positive gaze-head angle (gaze is ahead of head direction).

Footage from the Mobile Eye camera was manually digitised to determine the coordinates of five different spatial locations in each frame of video footage: the (i) ball, (ii) location of gaze, (iii) location of ball-release, (iv) bottom-left of the projection screen, and (v) bottom-right of the projection screen. The first three reference points were used to calculate three raw angles (in degrees) subtended at the eye relative to the initial direction at the moment of ball-release: the *ball angle*, *gaze angle*, and *head angle* (Fig. 1A). The final two reference points (bottom left and right of the projection screen) were used to calculate and correct for head rotation to ensure that all three angles were reported relative to global rather than local coordinates. The calculation of head angle required the assumption that the predominant head movement was rotation rather than translation; this is a good generalisation in the case of cricket batting, as the ball is directed towards the batter who will typically attempt to remain in-line with the directional path of the ball. Three relative angles were calculated to convey the comparative position of the three raw angles: the *gaze-ball angle*, *head-ball angle*, and *gaze-head angle* (Fig. 1B).

The head-mounted camera in the Mobile Eye gaze-tracking system moves commensurate with movement of the head. As a result, the location of the ball relative to the direction of the head was recorded and visualised using scatterplots of the x-y digitised coordinates of the ball for every video frame in a single trial. A tight cluster of coordinates within a single trial indicates that rotation of the head was tightly coupled to the ball, meaning the ball was kept in the same direction relative to the head.

In addition to the digitisation of the video clips, each clip was manually viewed frame-by-frame for two specific reasons. First, we sought to verify the presence and timing of any visual saccades that took place between the moments of ball-release and bat-ball contact. A saccade was recorded when there was a distinct change in the location of gaze that did not move commensurate with the path of the ball. Three types of saccades were recorded: (i) saccades to the location of ball-bounce, (ii) saccades beyond the location of ball-bounce, and (iii) other saccades not to or beyond ball-bounce (usually performed to ‘catch-up’ with the ball after gaze had lagged behind it). A high level of intra-tester and inter-tester reliability was found when coding the moment each saccade took place for all trials for a single participant (94.4% agreement for each; coding two-weeks apart for intra-tester reliability). Second, we sought to determine whether gaze was directed towards the ball at the moment of bat-ball contact. Although the measurement of gaze-ball angle would be desirable to do so, manual viewing was necessary for two specific reasons. First, the bat and ball were not visible in the Mobile Eye footage at the moment of bat-ball contact in some of the trials, particularly in many for the club-level batters, as they did not rotate their head downwards as much as the elite batters did (gaze, though, was generally still visible in the footage so it was obvious that gaze was not directed towards the ball in these instances). Second, for those trials where the bat and ball *were* visible at bat-ball contact, the speed of the ball and the frame-rate of the Mobile Eye meant that there was a large error in measuring the gaze-ball angle when the ball was very close to the batters. [The Mobile Eye captures video frames at 40 ms intervals. For a ball travelling at 33 m/s, the ball can be up to ±0.65 m from the ultimate location of bat-ball contact in the frame closest to contact. If bat-ball contact occurs 1.5 m from the eye, this equates to an error margin of ≈23 degrees of visual angle. As a result, this method was clearly not suitable for an accurate numerical assessment of gaze at bat-ball contact.] As a result, we manually viewed the footage to determine whether gaze was directed towards the ball at the moment it was hit. Specifically, from the observation of the frames immediately prior to and after bat-ball contact, we judged whether gaze was likely to have been within 4 degrees of the bat at the moment the ball was hit (a visual angle equating to 1 bat-width at a distance of 1.5 m from the eye). This method cannot determine whether the batters directly aligned their fovea (central vision) with the ball at the moment it was hit, but it does provide a clear differentiation of whether gaze was directed towards the location of bat-ball contact at the moment the ball was hit, or alternately whether vision was directed elsewhere (usually lagging behind the ball). This manual coding of gaze at bat-ball contact demonstrated a high level of intra-tester and inter-tester reliability (100% and 91% agreement respectively).

Only those trials where the participant swung their bat and made contact with the ball were included in the final analysis because batters tend to stop visually tracking the ball when they ‘leave’ deliveries deemed unsuitable to hit. Three trials were excluded from the analysis as a result of the Mobile Eye failing to record the location of gaze at some point during the trial.

### Statistical Analyses

Independent *t*-tests (two-tailed) were used to compare dependent variables between the elite and club-level batters. Alpha was set at.05 for all comparisons. Trials for each participant were used as individual observations for statistical analyses; this violates the assumption of independence of observations, but is consistent with previous work and has been deemed appropriate for the examination of small sample sizes [7], [32]. Levene’s test was used to check for the equality of error variances, and any violations were subject to the Welch-Satterthwaite method for adjusting the degrees of freedom to minimise the chance of Type I errors.

## Results

**Figure 2:**
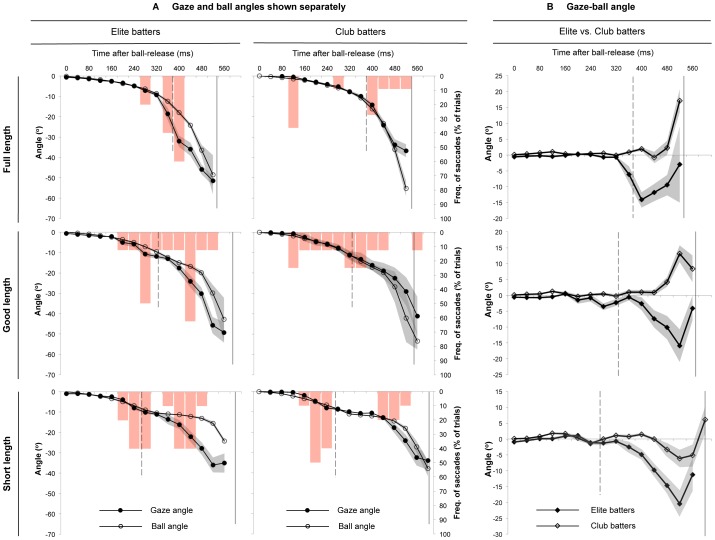
Direction of gaze relative to the ball. Panel A shows, for each combination of level of batting skill and location of ball-bounce, (i) the mean vertical gaze angle and ball angle (black lines with closed and open circles respectively), and (ii) for each time point, the proportion of trials where a saccadic eye movement was initiated (*frequency of saccades*; red columns). Panel B shows the mean vertical gaze-ball angle for each location of ball-bounce. Grey shaded areas represent SE across trials, broken vertical lines indicate the mean time of ball-bounce, and solid vertical lines indicate the mean time of bat-ball contact.

An examination of the direction of gaze relative to the position of the ball revealed that the elite batters kept their gaze either in alignment with, or ahead of the ball, irrespective of where the ball bounced (Fig. 2). In contrast, the gaze of the club-level batters was much more likely to be in alignment with, or *behind* the location of the ball. The club batters only directed their gaze ahead of the ball on a consistent basis when hitting the short length trials, and even in those trials it was only for a short period of time between ball-bounce and bat-ball contact. The statistical analysis of the gaze-ball angle confirmed that the elite batters directed their gaze further ahead of the ball than the club-level batters did (mean gaze-ball angle, Elite vs. Club [*Mean* ± *SD*] = −3.4±1.5 vs. 0.9±1.4 deg, *t*(56) = −10.9, *p*<.0001), and that they did so for a longer proportion of ball-flight (gaze at least 2 degrees ahead of ball, 30.5±11.3 vs. 8.2±9.9% of ball-flight, *t*(56) = 8.0, *p*<.0001).

The comparative analysis of the gaze and ball angles also highlights the presence and timing of the saccadic eye movements made when batters hit the ball. The rectangular columns in Figure 2 show how frequently saccades were initiated for each of the time points within the trials. These columns show that the elite batters tended to produce a single saccade in the full-length trials (reflected by a monophasic pattern for the frequency of the saccades), but that *two* saccades were produced in the good and short-length trials (reflected by a biphasic pattern). These groupings of saccades for the elite batters are mirrored by commensurate changes in the gaze angle relative to the location of the ball, in particular, at 360–400 ms after ball-release in the full-length trials, at 280 and 440 ms in the good-length trials, and at 240–280 and 400–440 ms in the short length trials. In contrast, there is much less consistency in the saccadic behaviour of the club-level batters, that is to say, the timing of their saccades is more evenly distributed across the different time periods throughout the trials. We revisit the saccadic eye movements shortly when considering the experimental trials on an individual basis.

The direction of gaze relative to the ball in the time-period immediately prior to bat-ball contact reveals an important differentiation between the elite and club-level batters. Figure 2B shows that the elite batters directed their gaze *ahead* of the flight-path of the ball immediately prior to bat-ball contact, whereas the gaze of the club-level batters tended to be *behind* the ball. The elite batters appeared to use a strategy that ensured they could ‘park’ their gaze ahead of the ball so that gaze could ‘lie-in-wait’ for the ball to arrive (this is best exemplified in Fig. 2A against the good and short length trials). Although the calculation of the gaze and ball angle was not always possible immediately prior to bat-ball contact, Fig. 2 clearly shows that the ball was looming towards the direction of gaze for the elite batters as the moment of bat-ball contact approached (i.e., so that the gaze-ball angle would be zero). In contrast, the club-level batters were much less likely to locate their central gaze ahead of the ball immediately prior to contact; only against the short-length trials did the club-level batters exhibit an ability to consistently do so. We return to a more thorough investigation of this issue shortly.

**Figure 3:**
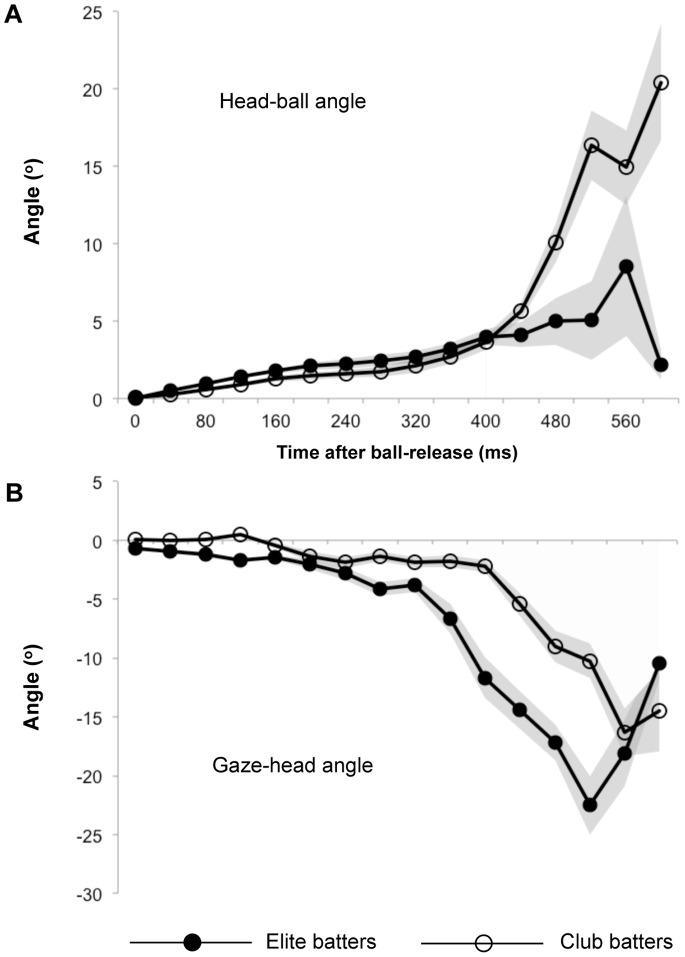
Mean head-ball and gaze-head angles. Comparison of (A) mean head-ball and (B) gaze-head angles for the elite and club-level batters. Gray shaded areas represent SE across trials.

An examination of the head-ball and gaze-head angles reveals that there were two specific strategies that the elite batters used which may have ensured they were better able to move their gaze ahead of the ball. First, the batters closely coupled the rotation of their head to the movement of the ball (Fig. 3A). The head-ball angle shows the elite batters had a tighter coupling between their head direction and the location of the ball (mean head-ball angle; 2.4±1.7 vs. 3.7±1.4 deg; *t*(56) = −3.2, *p* = .003), particularly in latter ball-flight when the head angle of the club-level batters lagged further behind the ball (maximum head-ball angle; 10.7±8.7 vs. 20.6±8.7 deg; *t*(56) = −4.2, *p*<.0001). Second, the elite batters moved their gaze further in advance of their head-direction (Fig. 3B). The gaze-head angle reflects the ability of the elite batters to make larger and earlier saccadic eye movements (minimum gaze-head angle, −24.1±8.2 vs. −14.1±7.6 deg, *t*(56) = −4.8, *p*<.0001; time of minimum gaze-head angle, 476±67 vs. 515±50 ms, *t*(56) = −2.5, *p* = .015, respectively). We now progress by concentrating on each of these two strategies in detail, doing so by considering the experimental trials on an individual basis.

### Head-ball Coupling

**Figure 4:**
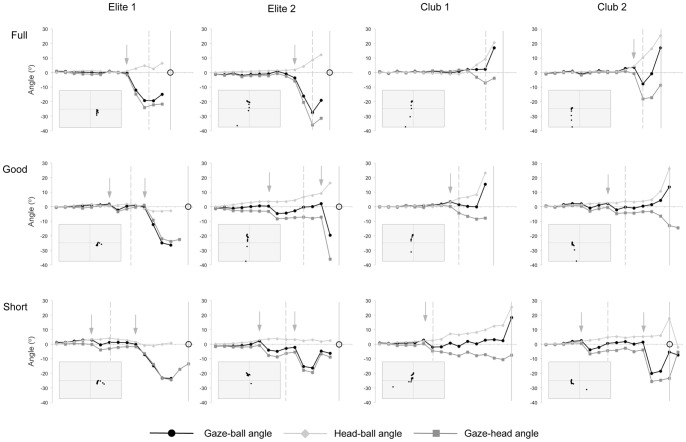
Exemplar trials for each batsman. Demonstration of gaze-ball, gaze-head, and head-ball angles in an exemplar trial for each location of ball-bounce for all four participants. Vertical arrows indicate predictive saccades. Broken vertical lines indicate the timing of ball bounce and solid vertical lines indicate the timing of bat-ball contact. Open circles highlight that an experimenter viewing Mobile Eye footage of the trial judged gaze to have coincided with the ball at the moment it was hit (see Method). Rectangular insets show the location (in x-y coordinates for each video frame) of the ball relative to the direction of the batter’s head (see Method).

The comparison of the three relative angles (gaze-ball, head-ball, and gaze-head) illustrates an important differentiation between the way that the elite and club batters appeared to track the ball: the elite batters closely aligned their *head* with the location of the ball, whereas the club-level batters more closely aligned their *eyes* with the ball. The elite batters coordinated their eyes and head in a fashion that minimised the discrepancy between the direction of the ball and head (head-ball angle<gaze-ball and gaze-head angles [mean absolute values]; *p*s <.001), whereas the club-level batters minimised the discrepancy between the direction of the ball and gaze (gaze-ball angle<head-ball and gaze-head angles [mean absolute values]; *p*s <.0001). This pattern of behaviour is illustrated in the individual exemplary trials shown in Fig. 4; of the three relative angles, the elite batters kept the head-ball angle closest to zero throughout the trials, where the club batters kept the gaze-ball angle closest to zero throughout the trials.

The coupling between the head and ball was visualised within each trial using plots of the x-y coordinates of the ball relative to the position of the head (Fig. 4 inset images). In these figures a tight cluster of coordinates reflects tight coupling between the ball and head such that the ball was kept in a consistent egocentric direction. To better understand this concept, one could consider the analogy of a ‘miner’s torch’. The light from a metaphorical torch, attached to the forehead of a batter with tight head-ball coupling, would shine on the ball from the moment of release through to, or very close to, the moment of bat-ball contact. In contrast, the light-beam from a batter with poor head-ball coupling would not remain on the ball. Accordingly, there is a tight cluster of head-centered ball coordinates in each trial for the elite batters (particularly for E1) that is less apparent for the club-level batters. Importantly, this head movement means that the ball would have remained close to central vision, particularly for the elite batters, if they simply kept their eyes still and only moved their head. That is to say, eye movements would not be necessary to accurately track the ball.

Interestingly, head-ball coupling was maintained even when the ball was in the peripheral vision of the batters. The gaze-ball angle (Fig. 4, main images) shows that central vision was located well in advance of the ball immediately following the saccades, yet tight head-ball coupling continued. It appears that peripheral vision and/or a memory-based expectation of the flight-path (potentially predicated on early ball-flight information) may have been helping, at least in these moments, to facilitate head-ball coupling.

### Saccadic Eye Movements

**Figure 5:**
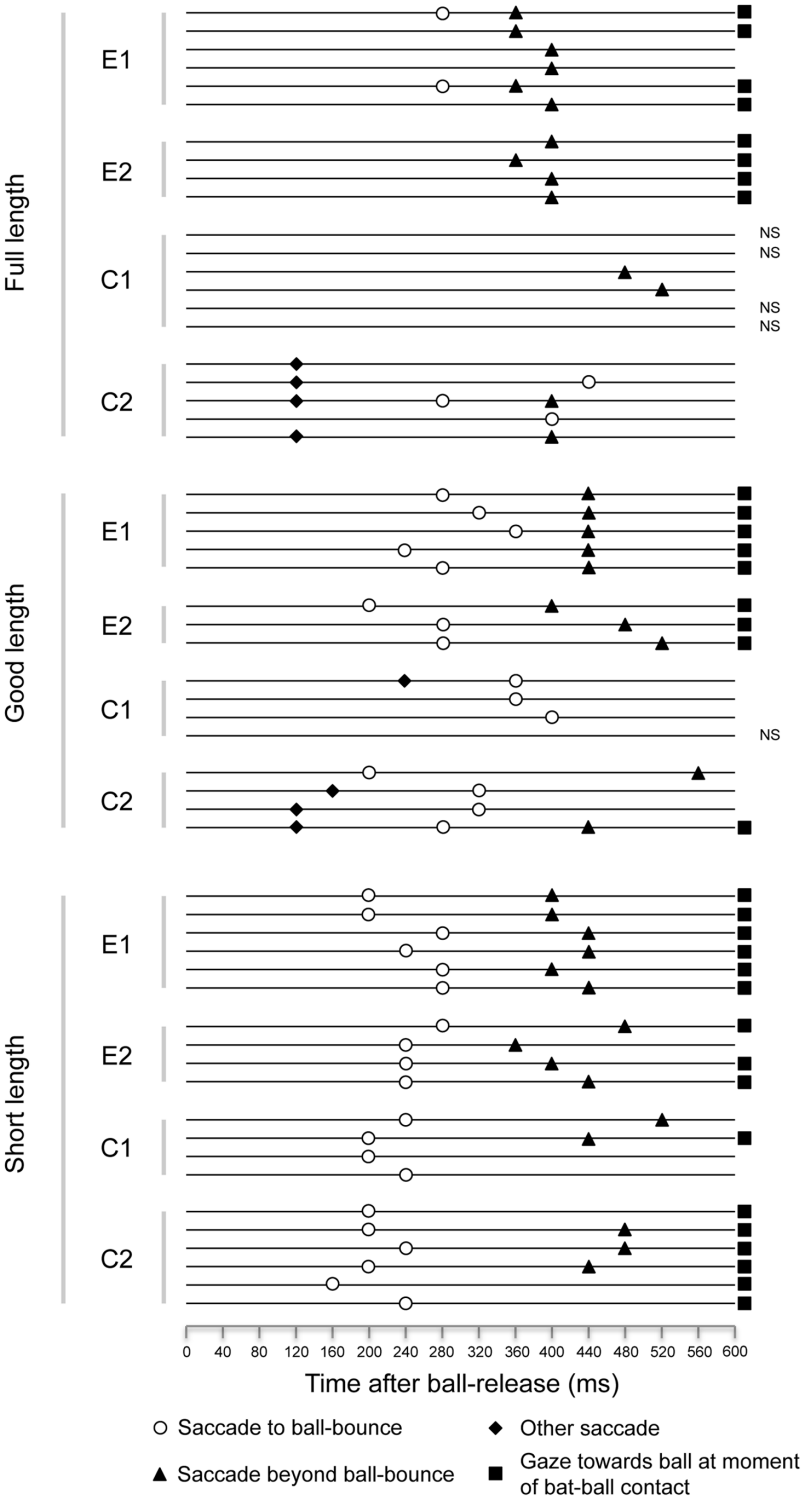
Saccadic eye movements. Horizontal lines represent the time course of each trial for the two elite (E1 & E2) and two club-level (C1 & C2) batters, showing (i) the presence and timing of saccadic eye-movements, and (ii) whether gaze coincided with the ball at the moment of bat-ball contact. NS indicates that no saccade took place in that trial.

The manual inspection of the individual trials confirms that the saccadic behaviour of the batters changed commensurate with their level of batting skill. The elite batters made not only larger and earlier eye movements [see also 7], but they also made *more* saccadic eye movements, ensuring that gaze was located further ahead of the ball for a greater proportion of ball-flight than it was for the club level batters (Figs. 4 & 5). Most interestingly, the elite batters often produced two distinct saccades: the first to predict ball-bounce, and the second to predict bat-ball contact (Fig. 5). Previous studies report a single saccade irrespective of where the ball bounced [6], [7], a finding consistent only with our lesser-skilled batters. In our study, two saccades were made by the elite batters on every good- and short-length trial, ensuring that gaze was judged to have coincided with the location of bat-ball contact for 100% of the good-length trials, and 90% of the short-length trials (Fig. 5). In contrast, the club batters made an initial saccade to ball-bounce in the good- and short-length trials (88% and 100% of trials respectively), but were much less likely to make a second saccade (only 25% and 50% of trials respectively). This resulted in them being substantially less likely to direct their gaze towards the bat as it hit the ball (13% and 70% of trials respectively). All batters typically made only one saccade against the full-length trials, though the elite but not club batters moved their gaze beyond ball-bounce to direct their gaze towards the bat as it hit the ball (Fig. 5; 80% of trials). The gaze of the club-level batters was judged to have lagged behind the location of bat-ball contact in each of the full-length trials.

## Discussion

This study provides a unique insight into how world-class batters achieve extraordinary levels of interceptive precision. The elite cricket batters exhibited two key differences in their visual-motor behavior when compared to the club-level batters: (i) a superior ability to couple the direction of the head to the movement of the ball, and (ii) eye movement strategies that ultimately predicted the location where the bat would make contact with the ball.

First, the ability to couple the head to the movement of the ball appears to be an important hallmark of expertise in batting. On one hand, this may not be all that surprising: cricket batters are often coached to move their head towards the line of the ball (so that it will be directly above the ball when it is hit), and batters are sometimes coached to rotate their head downwards so that the ball is hit when it is *under their nose.* However, even with these observations in mind, the remarkably precise head-ball coupling found for the elite batters in this study still comes as a surprise. The strength of the head-ball coupling leads us to question what may be the functional advantage of this behaviour. Visual-motor tasks like catching and hitting are understood to be controlled in an egocentric manner that is consistent according to self-centered coordinates [21], and so it may be an advantage to keep the target in a consistent direction relative to the head. In this sense, the elite batters may have learned to use their eyes to guide their head, ensuring their head direction is aligned with the position of the ball. [Incidentally, the relationship between the head direction and a target can be mapped onto neurons that respond to the spatial position of a target relative to the direction of the head, independently of where the eyes are looking [33], [34].] By reducing the location of the ball to a single egocentric direction, it is possible that the elite batters could derive a very simple, yet elegant, means of perceiving exactly *where* the ball will arrive relative to the direction of their head [35]. By knowing where the ball will arrive, they could simplify the hitting task to one where they must simply determine the time-to-contact necessary to successfully intercept the target [36], [37]. In contrast, the club-level batters were less capable of maintaining head-ball coupling, meaning they may be less certain of, and must otherwise predict, the future arrival point of the ball. Egocentric direction has been implicated in the control of other motor tasks, for instance, it can be used to control the guidance of walking when navigating towards a stationary goal [38], [39], and a skilled race-car driver may use their head direction to help the control of steering [25]. It may be that the head direction has an important role to play in the guidance of hitting movements like those performed by cricket batters.

Second, the elite batters seemed to produce consistent eye movement strategies that ensured gaze was directed towards the bat at the moment it made contact with the ball. This finding is in direct contrast to existing studies that suggest this is not the case, and some that suggest that it may not even be possible to do so [4], [7]. Land and McLeod [7] reported that the batters in their study “tracked the ball accurately for at least 0.2 s after the bounce, then more loosely tracked the ball on its final approach to the bat” and that the batters “lost the ball at the end of its trajectory” (p. 1342). They concluded “there seemed to be no systematic differences in the way the three batsmen tracked the ball after the bounce” (p. 1343). Our findings show otherwise, instead providing support for the anecdotal reports of Justin Langer and other elite batters who say they can watch the bat hit the ball [e.g., 4]. In fact, the elite batters appeared to do whatever was necessary to ensure that their gaze was directed towards the location of bat-ball contact: usually they made two predictive saccades, but even when they produced only one, they shifted gaze to the anticipated location of bat-ball contact rather than to ball-bounce [c.f., 7]. Evidently, it may be feasible to follow a coach’s direction to *watch the ball onto the bat*; unfortunately, though, it might be an aspiration consistently achievable by only a small minority of players.

Predictive saccades seem to play an important role in batting, though it is not immediately clear what that role may be. A number of previous studies have suggested that saccades may be produced because the ball moves too quickly to be tracked by the eyes [4], [6], [7]. Our data suggest that this is unlikely. The elite batters initiated saccades when the gaze-ball angle was low, showing that gaze was accurately aligned with the ball when the saccades were initiated. More importantly, the strikingly low head-ball angle highlights that the head itself was directed towards the ball throughout the majority of ball-flight. As a result, gaze would have been directed towards the ball if only the head were to move and the eyes were simply kept still relative to the head. Further support is evident from a recent study by Croft et al. [6]. Based on the assumption that saccades must be necessary because the ball moves too quickly to be tracked by the eyes, Croft et al. sought to establish the threshold ball velocity below which saccadic eye movements would no longer be necessary. They systematically varied the velocity of balls that were intercepted by skilled junior cricket batters but found that predictive saccades were produced irrespective of the velocity of the ball. In other words, they were unable to find a threshold speed below which saccades were no longer necessary. Evidently, saccades may not be required to compensate for a ball that is moving too quickly to be tracked by the eyes.

If predictive saccades are not required to compensate for ball-speeds that are too fast to be tracked by the eyes, then this of course leads us to ponder what might be the role of the saccades. We suggest three possibilities. First, it has been proposed that saccades may facilitate better gaze tracking after a discontinuity in the flight-path of a target [9]. The results of our study provide some support for this supposition, though the discontinuity might not need to be a change in direction *per se*, rather it can more simply be a change in the angular velocity of the target subtended at the observers eye. For the batters in our study, the direction of gaze did not need to change to track the ball after it bounced, only the angular *velocity* of the direction of gaze needed to change. In other words, the ball continued to move downwards in the field of view of the batter (i.e., the ball angle continued to decrease) irrespective of where the ball bounced (see the ball angles in Fig. 2; only for a short period after ball-bounce in the short length trials did the ball loom directly towards the observer’s eyes). This means that at no point were the batters required to direct their gaze downwards towards ball-bounce, and then back up again after the ball had bounced. Hence, the saccades may help to facilitate tracking after bounce, as they allow the eyes to avoid the change in velocity that would be necessary to accurately track the ball after it bounced. Second, it is possible that predictive saccades allow batters to better detect, and subsequently adapt to, *unexpected* changes in the flight-path of the ball after it bounces. While most spherical objects bouncing off the ground (or any other surface) can be expected to rebound in a relatively predictable manner, there will be times when unexpected deviations occur, for example, if the ball or the surface it rebounds from is not perfectly flat and/or regular, or if the ball is spinning before it hits the ground. In these cases the predictive saccade may direct the fovea towards the location of ball-bounce to ensure that gaze can quickly monitor for, and align itself with, any change in the direction of the ball after it has bounced. Finally, the saccades may play a functional role in detecting whether the anticipated and actual flight-paths of the ball are in agreement [e.g., 25]. Following a saccade to ball-bounce or bat-ball contact, peripheral vision could be used to monitor whether the ball is looming towards the direction of the fovea (i.e., the predicted future location of the ball) [40], [41]. It is not immediately clear which of these three (or possibly other) explanations best accounts for the role of predictive saccades; further work is necessary to systematically address how saccades facilitate interceptive success.

Previous work has argued that the portions of ball-flight where the direction of gaze coincides with the position of the ball should indicate the ball-flight information that is most critical for successful interception [7]. Accordingly, our results imply that early ball-flight, ball-bounce, *and* bat-ball contact may contain critical sources of information. However, there is considerable debate whether late ball-flight information can be used to alter a hitting action. For example, it has been shown that skilled cricket batsmen require at least 190 ms to adapt their bat-swing when a ball bounces in an unexpected fashion [13], and this finding is sometimes interpreted to be evidence that no useful changes can be made to the bat-swing during this period. However, Bootsma and van Wieringen [1] have persuasively argued that there should be a clear distinction between the visual-motor delay that is necessary for a performer to adapt to an *unexpected* error (as was the case in the study by McLeod [13]), and the delay necessary when using visual information to guide a movement as it *naturally unfolds*. It is reasonable to expect that the visual-motor delay may be considerably shorter for the continuous guidance of a movement, as is likely to be the case for the interceptive actions examined in our study. Patently the information at bat-ball contact was not necessary to make contact with the ball, as even the club-level batters managed to hit the ball on all of the trials we examined. Conceivably, though, by being better able to predict, and locate their gaze towards, the future location of bat-ball contact, the elite batters may be able to use their peripheral vision to facilitate continuous visual-motor regulation of the bat-swing as late as may be physically permissible [1]. It is known that anchoring vision ahead of a target can facilitate a better estimation of the moment of impact compared to what is possible if the moving target is fixated [8]. Cricket batters anecdotally report that they make fine adjustments to their wrist orientation in an effort to ensure the ball is directed away from opposition fielders when they hit it. Future studies may be able to uncover whether the ability to ‘park’ gaze at the anticipated location of bat-ball contact provides some form of functional advantage that may result in more efficacious hitting and/or a decreased likelihood that the batter misjudges the precision and accuracy of the interceptive action.

## References

[1] Bootsma RJ, van Wieringen PCW (1990) Timing an attacking forehand drive in table tennis. Journal of Experimental Psychology: Human Perception and Performance 16: 21–29.

[2] Regan D (1997) Visual factors in hitting and catching. Journal of Sports Sciences 15: 533–558. 9486432 10.1080/026404197366985

[3] Sarpeshkar V, Mann DL (2011) Biomechanics and visual-motor control: how it has, is, and will be used to reveal the secrets of hitting a cricket ball. Sports Biomechanics 10: 306–323. 22303783 10.1080/14763141.2011.629207

[4] Bahill AT, LaRitz T (1984) Why can’t batters keep their eyes on the ball? American Scientist 72: 249–253.

[5] Hubbard AW, Seng CN (1954) Visual movements of batters. Research Quarterly 25: 42–57.

[6] Croft JL, Button C, Dicks M (2010) Visual strategies of sub-elite cricket batsmen in response to different ball velocities. Human Movement Science 29: 751–763. 20031242 10.1016/j.humov.2009.10.004

[7] Land MF, McLeod P (2000) From eye movements to actions: how batsmen hit the ball. Nature Neuroscience 3: 1340–1345. 11100157 10.1038/81887

[8] Ripoll H, Fleurance P (1988) What does keeping one’s eye on the ball mean? Ergonomics 31: 1647–1654. 3229411 10.1080/00140138808966814

[9] Hayhoe MM, McKinney T, Chajka K, Pelz JB (2012) Predictive eye movements in natural vision. Experimental Brain Research 217: 125–136. 22183755 10.1007/s00221-011-2979-2 PMC3328199

[10] Pinder RA, Davids K, Renshaw I, Araujo D (2011) Manipulating informational constraints shapes movement reorganization in interceptive actions. Attention, Perception, & Psychophysics 73: 1242–1254. 10.3758/s13414-011-0102-1 21327746

[11] Beilock SL, Carr TH, MacMahon C, Starkes JL (2002) When paying attention becomes counterproductive: impact of divided versus skill-focused attention on novice and experienced performance of sensorimotor skills. Journal of Experimental Psychology: Applied 8: 6–16. 12009178 10.1037//1076-898x.8.1.6

[12] Gray R (2009) Intercepting moving objects: Fundamental principles learned from baseball. Reviews of Human Factors and Ergonomics 5: 114–139.

[13] McLeod P (1987) Visual reaction and high-speed ball games. Perception 16: 49–59. 3671040 10.1068/p160049

[14] Tyldesley DA, Whiting HTA (1975) Operational timing. Journal of Human Movement Studies 1: 172–177.

[15] De Oliveira RF, Huys R, Oudejans RRD, van de Langenberg R, Beek PJ (2007) Basketball jump shooting is controlled online by vision. Experimental Psychology 54: 180–186. 17725158 10.1027/1618-3169.54.3.180

[16] Benguigui N, Ripoll H, Broderick MP (2003) Time-to-contact estimation of accelerated stimuli is based on first-order information. Journal of Experimental Psychology: Human Perception and Performance 29: 1083–1101. 14640832 10.1037/0096-1523.29.6.1083

[17] Bongers RM, Michaels CF (2008) The role of eye and head movements in detecting information about fly balls. Journal of Experimental Psychology: Human Perception and Performance 34: 1515–1523. 19045990 10.1037/a0011974

[18] Gibson JJ (1966) The senses considered as perceptual systems. Oxford, UK: Houghton Mifflin.

[19] LaFont D (2007) Watch the ball? ITF Coaching and Sport Science Review 43: 11.

[20] Zaal FTJM, Michaels CF (2003) The information for catching fly balls: Judging and intercepting virtual balls in a CAVE. Journal of Experimental Psychology: Human Perception and Performance 29: 537–555. 12848325 10.1037/0096-1523.29.3.537

[21] Oudejans RRD, Michaels CF, Bakker FC, Davids K (1999) Shedding some light on catching in the dark: Perceptual mechanisms for catching fly balls. Journal of Experimental Psychology: Human Perception and Performance 25: 531–542. 10205865 10.1037//0096-1523.25.2.531

[22] De Wit MM, Van Der Kamp J, Masters R (2012) Distinct task-independent visual thresholds for egocentric and allocentric information pick up. Consciousness and Cognition 21: 1410–1418. 22868214 10.1016/j.concog.2012.07.008

[23] Milner AD, Goodale MA (1995) The visual brain in action. Oxford, UK: Oxford University Press.

[24] Ripoll H, Bard C, Paillard J (1986) Stabilization of head and eyes on target as a factor in successful basketball shooting. Human Movement Science 5: 47–58.

[25] Land MF, Tatler BW (2001) Steering with the head: The visual strategy of a racing driver. Current Biology 11: 1215–1220. 11516955 10.1016/s0960-9822(01)00351-7

[26] Müller S, Abernethy B (2006) Batting with occluded vision: an in situ examination of the information pick-up and interceptive skills of high- and low-skilled cricket batsmen. Journal of Science and Medicine in Sport 9: 446–458. 16713351 10.1016/j.jsams.2006.03.029

[27] Mann DL, Abernethy B, Farrow D (2010) The resilience of natural interceptive actions to refractive blur. Human Movement Science 29: 386–400. 20430464 10.1016/j.humov.2010.02.007

[28] Mann DL, Abernethy B, Farrow D (2010) Visual information underpinning skilled anticipation: the effect of blur on a coupled and uncoupled in-situ anticipatory response. Attention, Perception, & Psychophysics 72: 1317–1326. 10.3758/APP.72.5.1317 20601713

[29] Pinder RA, Renshaw I, Davids K (2009) Information-movement coupling in developing cricketers under changing ecological practice constraints. Human Movement Science 28: 468–479. 19339072 10.1016/j.humov.2009.02.003

[30] Mann DL, Abernethy B, Farrow D (2010) Action specificity increases anticipatory performance and the expert advantage in natural interceptive tasks. Acta Psychologica 135: 17–23. 20507831 10.1016/j.actpsy.2010.04.006

[31] Shim J, Carlton LG, Chow JW, Chae WS (2005) The use of anticipatory visual cues by highly skilled tennis players. Journal of Motor Behavior 37: 164–175. 15730949 10.3200/JMBR.37.2.164-175

[32] Bates BT (1996) Single-subject methodology: An alternative approach. Medicine & Science in Sports & Exercise 28: 631–638. 9148096 10.1097/00005768-199605000-00016

[33] Duhamel JR, Bremmer F, BenHamed S, Graf W (1997) Spatial invariance of visual receptive fields in parietal cortex neurons. Nature 389: 845–848. 9349815 10.1038/39865

[34] Galletti C, Battaglini P, Fattori P (1995) Eye position influence on the parieto-occipital area PO (V6) of the macaque monkey. European Journal of Neuroscience 7: 2486–2501. 8845954 10.1111/j.1460-9568.1995.tb01047.x

[35] McBeath MK, Shaffer DM, Kaiser MK (1995) How baseball outfielders determine where to run to catch fly balls. Science 268: 569–573. 7725104 10.1126/science.7725104

[36] Lee DN, Young DS, Reddish PE, Lough S, Clayton T (1983) Visual timing in hitting an accelerating ball. Quarterly Journal of Experimental Psychology 35A: 333–346. 10.1080/14640748308402138 6571315

[37] Lee DN, Young DS, Rewt D (1992) How do somersaulters land on their feet? Journal of Experimental Psychology: Human Perception and Performance 18: 1195–1202. 1431752 10.1037//0096-1523.18.4.1195

[38] Warren WH, Kay BA, Zosh WD, Duchon AP, Sahuc S (2001) Optic flow is used to control human walking. Nature Neuroscience 4: 213–216. 11175884 10.1038/84054

[39] Rushton SK, Harris JM, Lloyd MR, Wann JP (1998) Guidance of locomotion on foot uses perceived target location rather than optic flow. Current Biology 8: 1191–1194. 9799736 10.1016/s0960-9822(07)00492-7

[40] Herlihey TA, Rushton SK (2012) The role of discrepant retinal motion during walking in the realignment of egocentric space. Journal of Vision 12: 4, 1–11. 10.1167/12.3.4 22396464

[41] Held R, Freedman SJ (1963) Plasticity in human sensorimotor control. Science 142: 455–462. 14064442 10.1126/science.142.3591.455

